# Therapeutic and Nutraceutical Effects of Polyphenolics from Natural Sources

**DOI:** 10.3390/molecules27196225

**Published:** 2022-09-22

**Authors:** Mehtap Sahiner, A. Sanem Yilmaz, Buket Gungor, Yasmin Ayoubi, Nurettin Sahiner

**Affiliations:** 1 Bioengineering, Faculty of Engineering, Canakkale Onsekiz Mart University, Terzioglu Campus, Canakkale 17100, Turkey; 2 Department of Chemistry, Canakkale Onsekiz Mart University, Terizoglu Campus, Canakkale 17100, Turkey; 3 Department of Pharmacology, School of Medicine, Canakkale Onsekiz Mart University, Terzioglu Campus, Canakkale 17100, Turkey; 4 Department of Ophthalmology, Morsani College of Medicine, University of South Florida, 12901 Bruce B Downs B. Downs Blv., MDC 21, Tampa, FL 33612, USA; 5 Department of Chemical and Biomolecular Engineering, University of South Florida, Tampa, FL 33620, USA

**Keywords:** oxidative stress, cardioprotective, atherosclerosis, reactive oxygen species, anti-aging

## Abstract

The prevalence of cardiovascular disease, oxidative stress-related complications, and chronic age-related illnesses is gradually increasing worldwide. Several causes include the ineffectiveness of medicinal treatment therapies, their toxicity, their inability to provide radical solutions in some diseases, and the necessity of multiple drug therapy in certain chronic diseases. It is therefore necessary for alternative treatment methods to be sought. In this review, polyphenols were identified and classified according to their chemical structure, and the sources of these polyphenol molecules are indicated. The cardioprotective, ROS scavenging, anti-aging, anticancer properties of polyphenolic compounds have been demonstrated by the results of many studies, and these natural antioxidant molecules are potential alternative therapeutic agents.

## 1. Introduction

The emphasis on healthy diets and mindful eating patterns is gaining more importance among society. Until recently, concepts such as the Mediterranean-style diet, wine consumption habits, and dietary habits such as olive oil consumption were emphasized. Now, the word nutraceutical has begun to be added to these concepts. Nutraceuticals are natural substances such as vitamins, polyphenols, fiber, keratinoid, prebiotics, fatty acids, and bioactive peptides [1]. It is possible for these substances, which are defined as functional foods, to be included in foods. Polyphenols in this group, which are the secondary metabolites of plants, are found in high quantities in beverages such as tea, wine, grape juice, as well as in various fruits and vegetables [2,3]. Vegetable sources containing high levels of polyphenols include grape (*Vitis vinifera*) and red wine, mulberry (*Morus rubra*), peanut (*Arachis hypogaea*), and onion [4,5,6]. In addition, red pepper, eggplant, soybean, pear, garlic, carrot, and *Ziziphus* fruits are other foods that contain a significant amount of polyphenolic compounds [7,8,9]. Apart from plants, marine algae have been identified as important bioactive natural resources containing polyphenols, especially catechins, tannins and flavanol glycosides [10].

Environmental factors such as daylight, soil type, precipitation, and agricultural conditions have a major impact on the polyphenol content of natural products. For example, the biosynthesis of flavonoids as one of the most familiar members of polyphenols, is stimulated by light, and these polyphenols accumulate at different rates between the aerial tissues and other parts of the plants, even among fruits of the same tree [11,12]. In the extraction of plant-based polyphenols, methods such as maceration (by adding cold water to the plant and keeping it at room temperature for a period of a few hours to few days), percolation (passing the substances in the plants to the solvent by passing through a column), reflux, use of Soxhlet apparatus and boiling have been commonly used [13]. Depending on the employed methods, e.g., Soxhlet extraction, they may require the use of different solvents and/or high amounts of organic solvents, and therefore, the selectivity may be different for one polyphenolic component and may be high for other polyphenolic components. Therefore, novel extraction methods that are specific for certain polyphenolic need to be developed. Recently, supercritical fluid extraction, enzyme-assisted extraction, solid-phase extraction, or combinations of these similar techniques can afford some advantages [14].

Polyphenolic compounds can be in monomer, oligomer and polymer forms, or colored or colorless depending on pH. Although aglycone forms are not stable, polyphenolic compounds in plants are resistant to light, pH, or other conditions that can degrade them, due to glycosylation [7].

These natural substances have antioxidant, anti-inflammatory, anticancer, and antidiabetic properties and protective effects on cardiovascular and nervous system health [15,16]. It is thought that the consumption of foods containing polyphenolic components with a wide variety of protective and healing properties provides an alternative area that can be used for therapeutic purposes in many cases, including chronic diseases [17,18]. 

It is known that polyphenolics are closely related to the intestinal microbiota, which plays a major role in maintaining the healthy state of individuals or preventing diseases before they occur. Polyphenols can modulate the composition of the intestinal microbiota, thus affecting their own metabolism and bioavailability [19].

In this review, polyphenols are classified into four main groups according to their chemical structures. The mechanisms through which phenolic compounds act in chronic and acute diseases and the general pathology of cardiovascular disorders are explained. After the pathological processes are stated, the effects of polyphenols in each group are explained with various examples.

## 2. Classification of Polyphenols

**Figure 1:**
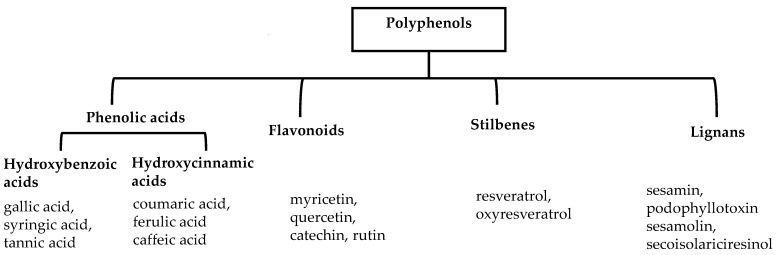
Schematic presentation of classification of polyphenols.

Polyphenols are a well-known group of phenolic systems characterized by at least two phenyl rings and one or more hydroxyl substituents [20]. Polyphenols can be found in all organs of plants. It is generally believed that flavonoids have weak resistance to diverse environmental stresses (heat, light, and oxidation). Polyphenols synthesized by plants can be simply divided into flavonoids and non-flavonoids. Polyphenols can be classified according to their chemical structure, whether they contain sugar or not, or by whether they are hydrolyzed or condensed. The sugar in polyphenol stabilizes the aglycone, increases its water solubility, and enhances its bioavailability and biocompatibility [21]. They can even be classified according to their resolution. Moreover, it can be classified based on its solubility or origin, for example, based on the plant or mammals that provide it [22]. As shown in Figure 1, polyphenols fall into four groups based on their chemical structure: phenolic acids, flavonoids, stilbenes, and lignans.

### 2.1. Phenolic Acids

Phenolic acids contain an aromatic benzene ring and a carboxylic acid group as shown in Figure 1. Gallic acid, chlorogenic acid and coumaric acid are the most known and widely used phenolic acids. They provide organoleptic properties to foods [23]. Fruits, vegetables, and beverages are good sources of phenolic acid. Adding vegetables to people’s diet as a significant source of phenolic acids will probably bring about significant health benefits. [23]. Phenolic acids are of interest to pharmacologists as well as food engineers. They are recognized as components of nutraceutical products and are gaining more and more attention every day. They are divided into two types as hydroxybenzoic acid derivatives and hydroxycinnamic acid derivatives. Red fruits, onions, and black radish generally contain hydroxybenzoic acids [24]. Gallic acid, protocatechuic acid, syringic acid, and ellagic acid are examples of hydroxybenzoic acid derivatives. 

Gallic acid (GA), 3,4,5-trihydroxy benzoic acid, is widely used as a reference substance in antioxidant tests [25,26]. GA is also a natural compound, and many fruits, nuts, and flowers (especially green tea and red wine) contain abundant amounts of GA [15]. GA has also been reported for its antioxidant, anticancer, neuroprotective capacity, anti-inflammatory properties, cardiovascular protective, hepatoprotective and gastroprotective activities [27]. GA has also been studied for anticancer properties [28] and for an antidiabetic effect [29]. Tannic acid is the most widely researched hydroxybenzoic acid. It is abundant in acorn and is inexpensive. Tannic acid hydrolyzes in acidic residue to form 10 molecules of gallic acid [30]. Due to its antimicrobial, antioxidant, anticancer properties, it is examined in biomedical materials [30]. In wound dressing materials, tannic acid is used as a coagulant because of its ability to clot blood [31].

Ferulic acid, caffeic acid, chlorogenic acid, sinapic acid, rosmarinic acid, isoferulic acid, and coumaric acid are included in the hydroxycinnamic acid subgroup. Prune and coffee are the source of phenolic acids. 

Rosmarinic acid (RA) is an example hydroxycinnamic acid. It is a known for its antioxidant and antidiabetic properties and is found in many plants including mint, rosemary, and basil [32]. There many research reports that detail the use of RA as a biomedical compound for treatment of organ injuries and in ocular delivery [33]. For type 2 diabetes mellitus, it has an effect on reducing the amount of glucose in the blood, as it inhibits the work of α-glucosidase and α-amylase enzymes that are involved in the breakdown of disaccharides [34]. RA also fights the complications of diabetes resulting in progressive organ damage via its anti-inflammatory and antioxidant activities [33]. The chemical structures and some of the subgroups of polyphenols are given in Table 1.

### 2.2. Flavonoids

The 15-carbon skeleton is the structural basis of polyphenolic compounds called flavonoids [35]. Rutin, quercetin, naringin, myricetin, catechin hesperidin, naringenin, isosakuratenin, and heridictyol are the other examples of flavonoids [36]. 

Naringin (NR) is the form of naringenin that is substituted with two glucose moieties [21], and the chemical structure is given in Figure 1. The sugar serves to stabilize the aglyocone, increasing the biocompatibility [21]. NR is abundant in grapefruit and is one of the components that gives grapefruit a bitter taste. NR exhibits antioxidant, anti-inflammatory, anti-apoptotic, anticancer, antimutagenic, and redox protective properties. It also reduces cholesterol levels, relieves neurodegenerative disorders, improves patients with diabetes mellitus, reduces osteoporosis, and alleviates rheumatoid arthritis [37].

Myricetin, 3,5,7-Trihydroxy-2-(3,4,5-trihydroxyphenyl)-4H-chromen-4-one is a flavonol. It is found in vegetables such as tomatoes, fruits such as oranges, nuts, berries, tea, and red wine. Flavonoids are divided into subclasses such as anthocyanins, flavonols, flavones, and anthocyanidin [20]. 

Plants contain color flavonoids called anthocyanins, which are commonly found in flowers. In addition to pollination and seed dispersal, plants display pollination and seed dispersal in their fruits and flowers. These components are also involved in a host of defense mechanisms against pathogens and herbivores while also contributing to the photosynthesis process. Pelargonidin, cyanidin, peonidin, delphinidin, petunidin, and malvidin are the most common anthocyanidins found in plants [35]. It is commonly studied to determine the total anthyocin amount of plants or some composite fabricated by extraction from plants [38]. During storage, anthocyanins’ colors change depending on pH, protonation, and hydration reactions. Cyanidin, malvidin, and chalcones are well-known and studied antocyanin structures [38].

### 2.3. Stilbenes

Two aromatic rings are linked by an ethylene ring in the stilbene, which has a backbone of C6–C2–C6 [39]. Resveratrol and oxyresveratrol are some samples of the stilbenes [40]. Oxyresveratrol has been shown to have inhibitory activity on Trichophyton rubrum, which causes fungal infections such as tinea pedis, and it has been emphasized that it has the potential to reduce the use of antibiotics as adjuvant therapy with its antimicrobial activity [40]. The skin of grapes, blueberries, raspberries, mulberries, and peanuts is the source of stilbenes. Resveratrol is widely found in red wine and grape juice. 

### 2.4. Lignans 

Lignans represent a nonflavonoid class featuring two propylbenzenes. Lignans are found in legumes, grains, seeds, and vegetable oils; they are mainly found in their free forms. Flaxseeds are excellent sources of lignans. Lignans are structures that protect plants against herbivores. They are generally insoluble. Sesamin and sesamolin are kinds of lignin molecules found in sesame seeds. The other known plant sources of lignans are secoisolariciresinol, podophyllotoxin and hydroxymatairesinol. Hydroxymatairesinol is a lignan found in Norway spruce [41]. Hydroxymatairesinol has been observed in Parkinson’s disease, which is a chronic neurodegenerative disorder, to slow down its progression in rat models [41]. It has been determined that it has anticancer properties in prostate cancer [42]. Pinoresinol is another lignan molecule that has hepatoprotective effects [43]. 

## 3. Cardioprotective Effects of Polyphenolic Compounds

Cardiovascular diseases (CVD) are a group of disorders that are characterized by heart and blood vessels and include coronary heart disease, cerebrovascular disease, congestive heart failure, rheumatic and congenital heart diseases. transient ischemic attack, or stroke, and carry a mortality and morbidity rate [44]. According to the calculations of the World Health Organization in 2016, CVD constitutes approximately 30% of death causes worldwide [45] and is considered to be the first cause of chronic, non-communicable deaths in adults. Deaths due to heart attacks and stroke constitute a large part of this calculated percentage [44]. The dramatic changes in the frequency of symptoms of CVDs in different age groups, both genders, and all races have been alarming in recent years. Although mortality rates vary according to income distribution, demographic population, diet, and lifestyle, it is among the diseases with the highest prevalence and incidence all over the world [46,47]. Risk factors for the development of CVD include long-term smoking, diabetes mellitus, unhealthy diet, intense stress, presence of CVD in family history, arterial hypertension, abnormal blood lipids, sudden paralysis, high cholesterol, obesity, and sedentary life [44,48]. It is known that multiple factors affect pathogenesis, but it is generally caused by atherosclerosis, which is known to occur as a result of oxidative stress and chronic inflammatory conditions [48].

Atherosclerosis is a degenerative process of the arteries characterized by thickening and hardening of the innermost intimal layer of the arterial walls. It is the most common pathological factor involved in the development of CVD. The healthy endothelial cells release nitric oxide (NO) to induce vasodilation, and contain anticoagulants, antiplatelet receptors, and structures that prevent thrombus formation, thus preventing unwanted aggregation of platelets and regulating vascular tone [49]. Nitric oxide is known to prevent the oxidation of LDL cholesterol, and if there is endothelial dysfunction, decreasing production of NO and impaired NO activity leads to consequences promoting atherosclerosis and is present in conditions such as vasoconstriction, smooth muscle cell proliferation, and oxidative stress.

Atherosclerotic plaque develops with an “active biological environment” such as interaction of endothelial dysfunction, vascular smooth muscle cell migration, lipid deposition (LDL oxidation), calcification, matrix turnover, immune response and inflammatory responses [47,50,51]. Hypertension, or high blood pressure, is one of the leading causes of cardiovascular diseases [52]. Hypertension affects both the cardiovascular and cerebrovascular systems. It has been reported that the incidence of hypertension increases with age and, if not controlled, leads to the risk of heart attack and stroke in patients [53]. 

**Figure 2:**
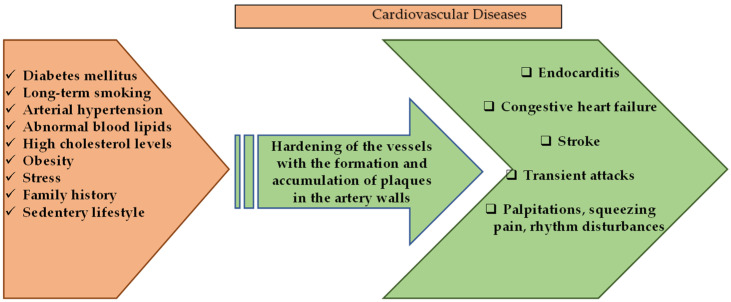
Schematic presentation of cardiovascular diseases and general factors involved in their pathology.

Cardiovascular diseases and general factors involved in their pathology are given in Figure 2.

Figure 2 demonstrates several genetic-based factors and many environmental factors involved in the pathological process that results in the emergence of cardiovascular system diseases. Many of these risk factors can be regulated by changes in the lifestyles of individuals [54,55]. Natural-based compounds are of interest in disease-prevention approaches and in the regulation of patients’ lifestyles, whose cardioprotective properties have already been proven or are currently being tested for effectiveness [55,56].

In addition to factors that cannot be changed, such as gender, genetic predisposition, and age, cardiovascular diseases also affect many external factors that can be controlled. The most important behavioral risk factors of cardiovascular diseases are unhealthy diet and physical inactivity. Among these, the way of nutrition and efforts to improve lifestyle habits sometimes provide a high protective effect against cardiovascular disease [54,55,57,58,59]. Alternative treatment methods are being developed for CVD, which has a high prevalence and incidence, and the consumption of functional foods and supplements has been on the agenda of clinical institutions in recent years [54].

As an example of the relationship between diet and CVD, excessive saturated fat diet increases the plasma LDL levels and subsequent risk of atherosclerosis [58]. Interest in new areas in the field of pharmaceutical and dietary strategies has started to minimize CVD risk as much as possible. Epidemiological studies have indicated that a high flavonoid content diet is positively related to lower incidence of coronary artery disease, hypertension, stroke, and other vascular diseases [60,61,62,63].

The protective effect of polyphenols on the cardiovascular system is due to their ability to delay the development and progression of early atherosclerotic lesions [54,57,58]. Another important mechanism of action is thought to be the regulation of NO release and antioxidant and anti-inflammatory properties of polyphenols [54,60].

The Mediterranean diet includes vegetables, fruits, bread, and other cereals, legumes and oilseeds, a moderate amount of alcohol consumption, and olive oil with a high content of polyphenolic compounds. It has been noted that this type of diet has a clear effect on reducing CVD risk factors and preventing cardiovascular diseases. The advantages of increased plasma HDL-C and decreasing LDL oxidation were seen through the controlled consumption of red wine [54,55,59]. In one of the studies evaluating the relationship between the Mediterranean diet and the risk of CV events, an inverse relationship was found between total polyphenol intake and CVD risk [64,65]. The effects of an herbal preparation consisting of procyanidin, daidzein, genistein, flavone, resveratrol on atherosclerosis in postmenopausal women without cardiovascular disease was investigated [66]. It was found that this preparation significantly suppressed the formation of atherosclerosis in individuals and could be a potential agent to slow the progression of existing plaques [66]. 

### 3.1. Phenolic Acids

An herbal combination with high polyphenol content composed of caffeic acid, chlorogenic acid, p-coumaric acid, and ferulic acid was administered to rabbits with myocardial cell damage, and it was emphasized that it could be used as an alternative treatment due to its synergistic cardio-protective potential [67]. In the study comparing the efficacy of administering a ferulic acid and aldose reductase inhibitor, which is the standard treatment, proved to alleviate insulin resistance and hypertension and mediated the restoration of endothelial-dependent relaxation by improving the bioavailability of NO [68]. Cardiac and kidney lesions, including oxidative stress, inflammation, vascular occlusion, and erosion of epithelial cells, were observed in rats that had been given the endocrine-disrupting chemical Bisphenol A (BPA). The addition of gallic acid into the BPA was found to mitigate the toxicity caused by the BPA [69]. 

The cardioprotective effects of tannic acid are thought to be mainly due to the reduction of myocardial oxidative stress, inhibition of inflammation, reduction of apoptosis, and increase in NO levels [70,71,72,73]. The potential cardioprotective properties of tannic acid were investigated in rabbits in a case of isoproterenol-induced myocardial ischemia [70]. Tannic acid has been proven to effectively heal ischemic damage. This therapeutic effect has been associated with inhibiting inflammation, providing endothelial protection, scavenging reactive oxygen species, reducing cell apoptosis and many other properties [70]. Tannic acid can reduce collagen accumulation on isoproterenol-induced myocardial tissue morphology, thereby reducing and healing myocardial mitochondrial damage [71]. It has been proven that tannic acid can target elastin stabilization in cardiovascular implants, rendering it enzymatically resistant [72]. Tannic acid at a concentration of 0.05% has been found to have minimal cytotoxic effect after 4 weeks of infarction, increasing the collagen content and significantly reducing infarction dilation. This treatment may attenuate adverse cardiac remodeling after acute myocardial infarction [74]. 

Another phenolic acid, caffeic acid, has cardioprotective properties, and relevant utilization and function from the literature are given in Table 2. During hypoxia or when exposed to uremic toxins, caffeic acid increased proliferation and angiogenesis as well as inhibited leukocyte adhesion and endothelial cell apoptosis [75].

### 3.2. Certain Cardioprotective Effects of Flavonoids

The most common flavonoids found in grapes are anthocyanins, flavonols, flavanols, dihydroflavanols, and proanthocyanidins. The study has shown that proanthocyanidins in grape seeds have the capacity to reduce or remove reactive oxygen radicals in the reperfused myocardium after ischemia, thereby leading to a recovery of the damage caused by reperfusion, a decrease in the incidence of arrhythmia, and a significant improvement in postischemic cardiac function [76]. Another study reported that the administration of grape polyphenol concentrate to rats with doxorubicin-induced cardiotoxicity resulted in a cardioprotective effect, possibly due to the increased level and activity of antioxidant enzymes [77]. In a study conducted in people at risk for type 2 diabetes, it was concluded that consumption of blueberry juice tends to improve systolic blood pressure through nitric oxide production [78]. 

The effect of a long-term quercetin diet on inflammation and physiological heart performance in dystrophic hearts were investigated [79]. According to the results, a quercetin-rich diet intake can be a potential treatment option for the complications of cardiac dystrophy. A medicinal plant, Thraatchathi Chooranam, containing various highly polyphenolic compounds such as gallic acid, quercetin, ellagic acid, galangin and naringenin provided a stabilizing, radical scavenging and protective effect on the cardiac membrane [80]. 

Epigallocatechin-3-gallate (EGCG), which is an important polyphenolic component also found in green tea, is thought to be cardioprotective against ischemia-reperfusion due to its antioxidant properties [81,82,83]. EGCG contributed to the heart’s adaptation to excessive pressure loading during the hypertrophy phase [84]. For this reason, it has been stated that EGCG can be a potential agent that can prevent heart failure due to cardiac hypertrophy [81]. EGCG can inhibit apoptosis by activating a certain pathway and shows a cardioprotective effect against ischemic damage [85]. Oral epigallocatechin-3-gallate pretreatment is thought to be an alternative cardioprotective application to prevent perioperative cardiac dysfunction during surgery [83]. 

The study examined increased cardiac toxicity associated with exposure to air pollution, and reported that feeding the rats that were exposed to air pollution for a long time dark chocolate containing high polyphenols downregulated myocardial inflammation genes and increased antioxidant and cardioprotective genes [86]. After consuming a fatty diet, drinking cocoa resulted in an increase in HDL and a decrease in LDL [87]. In addition, cocoa has been found to alleviate the effect of inflammation. 

A dietary supplement containing the flavonoids of apigenin, kaempferol, and luteolin improved glucose tolerance and fat accumulation, as well as plasma triglyceride and total cholesterol levels, and reduced heart damage [88]. Limonin has been found to significantly lower the level of apolipoprotein B(Apo-B), a potential marker for coronary artery disease, and this effect is greater than that of other citrus flavonoids [89]. 

Myricetin contributes to the vitality of human vascular smooth muscle cells and the function of enzyme activity [90]. Furthermore, dihydromyricetin exerts an anti-inflammatory effect by reducing the release of interleukins, which are inflammatory agents, and prevents increases in the harmful effects of reactive oxygen species in the process of atherosclerosis [91,92]. 

### 3.3. Stilbenes

The effects of resveratrol and red wine in vivo (in hypercholesterolemic rabbits) and in vitro (on platelets isolated from normotensive healthy male volunteers) were investigated. It has been reported that resveratrol can inhibit platelet aggregation both in vitro and in vivo, possibly due to the cardioprotective effects of the polyphenol [93]. There was a significant increase in lipid levels in rabbits fed high-cholesterol diets, and they stated that hypercholesterolemia increases the susceptibility to platelet aggregation and has a risk of playing a role in myocardial infarction. The polyphenolic compounds found in red wine decreased intracellular cholesterol levels [94]. In an experimental study on hypertensive rats, a significant increase in HDL levels and a decrease in blood pressure were found [95]. 

Many studies show that resveratrol is a potential cardioprotective agent [96,97,98,99]. The effect of resveratrol on LDL oxidation and the free radical scavenging activity of the compounds on rat hearts was evaluated, and astringinin showed stronger cardioprotective activity than resveratrol in ischemia-reperfusion injury, and they thought that this was due to its high water solubility [97]. Ungvari et al. (2007) confirmed that resveratrol partially exerts antioxidant effects and plays a regulatory role in the vascular expression of some enzymes [96]. They stated that enzyme regulation shows antiapoptotic activity in the cardiovascular system in case of ischemia-reperfusion pathology. Resveratrol preserved cardiac function, suppressed the expression of inflammatory response-initiating genes, and had a significant ameliorating effect on myocarditis [98]. Resveratrol treatment was found to attenuate induced cardiomyocyte apoptosis and reduce toxicity on the heart by suppressing stress-induced overexpression [99]. In addition, resveratrol can protect the heart from doxorubicin damage with its antioxidant properties [100]. A significant reduction in gene conjugate required for autophagic expansion and termination in the group receiving resveratrol supplements was found. During myocardial stress, autophagy, an adaptive process in which damaged cellular components are removed or recycled, is cardioprotective. Resveratrol supplementation has been found to regulate autophagy and prevent endothelial dysfunction and cardiac remodeling in ischemic myocardium [101]. 

Resveratrol provides regulatory effects on free fatty acid oxidation, glucose homeostasis, glucose utilization, myocardial metabolic enzymes, and energy metabolism [102]. Resveratrol can exert a protective effect by inhibiting endoplasmic reticulum stress and reversing apoptotic pathway proteins’ expression in cardiomyocyte hypertrophy. Partially beneficial effects of resveratrol, which may be a new strategy for the treatment of cardiac hypertrophy, have also been reported in studies [103]. The curative effect of resveratrol in cardiac hypertrophy is through expression regulation as a result of breast cancer type 1 sensitivity protein inhibition [104]. 

In ischemia-reperfused rats, piceatannol slows the inactivation of I(Na) and inhibits arrhythmias [105]. Piceatannol can prevent cardiomyocyte cell damage caused by oxidative stress by activating superoxide dismutase, decreasing creatine kinase, and reducing lactate dehydrogenase production [106].

### 3.4. Lignans

Cardiotoxicity was induced in rabbits pre-treated with flaxseed, and the myocardium was examined histopathologically. A decrease in myocardial necrosis and inflammation as well as improvement in hemodynamic and biochemical markers were detected in the flaxseed-fed group. The antioxidant effect of flaxseed was found to contribute to its cardioprotective effect [107]. A similar result was obtained in the study, and it was suggested that the cardioprotective effect was due to the alpha-linolenic acid content [108]. It has been proven that lignan concentrate (flax lignan concentrate (500 mg/kg) and omega-3-fatty acid (1 mL/kg)) provide an antihyperlipidemic effect by normalizing lipid levels, and exerts an anti-inflammatory anti-apoptotic effect by affecting many parameters such as tumor necrosis factor-α level [109]. In another study, rats with experimental renal hypertension were divided into groups and administered angiotensin converting enzyme inhibitor (ACE inhibitor) or flax lignan concentrate. Flax lignan concentrate was observed to significantly reduce arterial blood pressure, similar to the standard ACE inhibitor. In addition, its antioxidant activity was shown to reduce histopathological damage in heart and kidney and to restore abnormal lipid profiles [110]. In addition, the consumption of lignan containing flaxseed and flax oil in moderate amounts causes a significant decrease in LDL cholesterol levels, which play a role in the pathogenesis of atherosclerosis [111]. Therefore, they may have an important role against ischemic heart disease. The protective effects of natural products containing polyphenolics on cardiovascular system health are given in Figure 2. 

**Figure 3:**
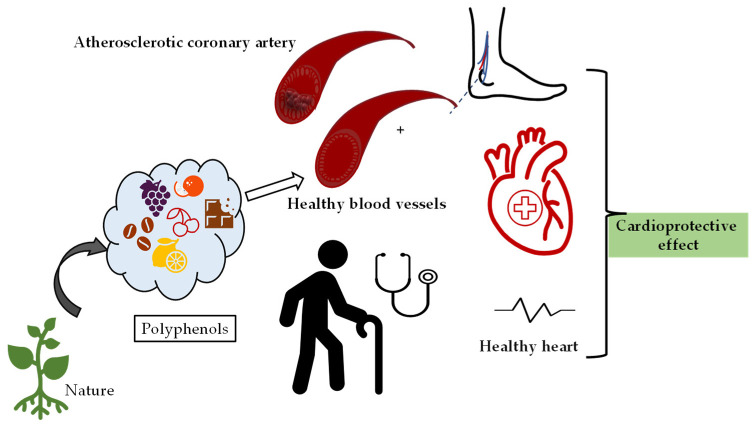
Demonstration of the protective effects of natural products containing polyphenolics on cardiovascular system health.

As can be seen in Figure 3, consumption of foods containing polyphenols has a boosting effect on cardiovascular health and plays a role in the regulation of heart rhythm and blood pressure against many factors such as aging.

Table 2 summarizes some of literature details on the application of some of polyphenols for cardiovascular diseases. 

**Table 2:** Some of the polyphenol efficacies, working concentration, and target of each substance on cardiovascular diseases.

Study Design	Outcomes	Ref.
Caffeic acid (100 nM and 1 μM) human umbilical vein-derived endothelial cells (HUVEC)	Caffeic acid increased proliferation and angiogenesis, inhibited leukocyte adhesion and endothelial cell apoptosis under hypoxia or by the uremic toxins’ conditions	[111]
Caffeic acid phenethyl ester (CAPE) 30 mg/kg/day administered by oral gavage for 6 weeks. Streptozotocin induced diabetes-induced atherosclerosis in rat model	CAPE abolished the diabetes-associated atherosclerotic changes by improving important functional and structural disorders in vessels. CAPE alleviated the elevation in systolic and diastolic BP	[112]
High fructose (HFCS) induced diabetic rats’ subacute CAPE administrations (50 μmol/kg/day intraperitoneally for 2 weeks	CAPE ameliorated the elevation in blood pressure, vascular damage, and it increased eNOS levels. CAPE lowered homocysteine and cholesterol levels	[113]
Atherogenic diet (Ath)-induced rat model administrations with caffeic acid 50 mg/kg, p.o.	CA ameliorated lipid profile and reduced the oxidative stress level. In aorta revealed reduction of the atherosclerotic lesions	[114]
Dietary 10% flaxseed content 1.37 mg/g SDG, (the lignan secoisolariciresinol diglucoside) LDL receptor-deficient mouse (LDLrKO) fed a cholesterol-supplemented diet and an increase in atherosclerotic plaque formation	Flaxseed lowered plasma cholesterol levels and saturated fatty acids, increased plasma ALA levels, and inhibited plaque formation in the aorta and reduced the inflammatory markers (IL-6, mac-3, and VCAM-1)	[115]
A 40 g/day of ground flaxseed administered Sixty-two men and post-menopausal women (LDL-C between 130 and 200) mg/dl 10 weeks	Flaxseed lowered LDL-C short lived and did not affect inflammation or oxidative stress	[116]
Low-density lipoprotein receptor knockout (LDLR−/−) mice with 170 g/kg sesame oil diet 3 months of feeding	Reduced atherosclerotic lesion formation, plasma cholesterol, triglyceride, and LDL cholesterol levels. Anti-inflammatory property (reduced inflammatory cytokines, such as MCP-1, RANTES, IL-1a, IL-6, and CXCL-16)	[117,118]
Sesamol 50 mg/kg orally for 6 weeks (DOCA)-salt-induced hypertensive rats	Decreased systolic and diastolic blood pressure and lipid peroxidation and enhanced the antioxidant activity. Hypertensive rats showed cardiac muscle fiber rupture and mononuclear infiltration, but Sesamol 50 mg/kg group heart showed to near-normal architecture	[119]
Isoproterenol treated myocardial infarcted rats, pretreated with gallic acid (15 mg/kg) daily for a period of 10 days	Prevented the changes in the activities of cardiac marker enzymes (CK-MB and LDH), reduced the levels of lipid peroxidation products (LPO), glutathione and lysosomal membrane damage	[120]
Rats were infused with AGEs (advanced glycation end products play a role development of cardiovascular disorders) and then treated with gallic acid (GA) by oral gavage daily at a dose of 25 mg/kg BW/day for 30 days	AGEs induced cardiac fibrosis and augmented oxidative stress in the heart tissues. GA prevented the upregulation of pro-fibrotic genes and ECM proteins (↓TNF-α, TGF-β, MMP-2 and -9 expression). GA treatment effectively prevented cardiac remodeling	[121]
NG-nitro-L-arginine methyl ester (L-NAME)-induced hypertensive mice treatment with gallic acid 100 mg/kg per day by daily intraperitoneal injections 3 or 8 weeks	GA attenuated cardiac fibrosis and remodeling, reduced the expression of histone deacetylase 1 (HDAC1) and 2 (HDAC2). GA lowered the elevated SBP	[122]

## 4. Protective Effects of Polyphenolic Compounds on ROS-Induced Oxidative Stress

Reactive oxygen species (ROS) are molecular oxygen byproducts that occur as a result of normal metabolism (such as oxidation–reduction reactions) in mitochondria and other cell organelles [123,124]. Disruption of the balance between these radicals and antioxidant molecules (endogenous-exogenous) in biological systems describes the state of oxidative stress. After prolonged oxidative stress, physiological impairments such as peroxidation (oxidation of unsaturated molecules) of membrane lipids occur. In addition, highly active organic molecules change important structural proteins non-enzymatically. The increase in ROS in tissues causes changes in the functions of intracellular proteins, resulting in DNA structural damage and cell damage [123,124,125]. In addition to the fact that ROS can occur due to natural factors (such as aging), ischemia-reperfusion damage, exposure to ionizing radiation and chemical agents negatively affect this process [126,127].

**Figure 4:**
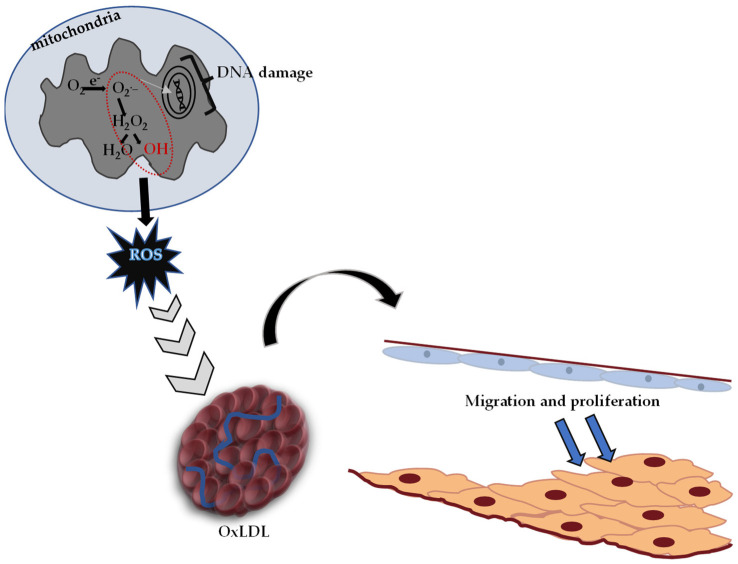
Reactive oxygen species (ROS) causing oxidative stress, oxidized low-density lipoprotein (OxLDL) and atherosclerotic plaque formation (adapted from ref. [129]).

It is known that oxidative stress and the DNA damage caused by it play an important role in the formation process of diabetes, cancer, atherosclerosis, heart attack, stroke and inflammatory disorders [128]. Reactive oxygen species (ROS) causing oxidative stress, low-density lipoprotein (LDL) oxidation and atherosclerotic plaque formation are given in Figure 4.

Toxic effects caused by free oxygen radicals, which are produced in significant amounts at the cellular level, are normally tried to be eliminated by the antioxidant defense system in the body. In cases where the antioxidant defense is insufficient, oxidative stress occurs, and Figure 4 shows the pathological process characterized by hardening in the arteries.

**Figure 5:**
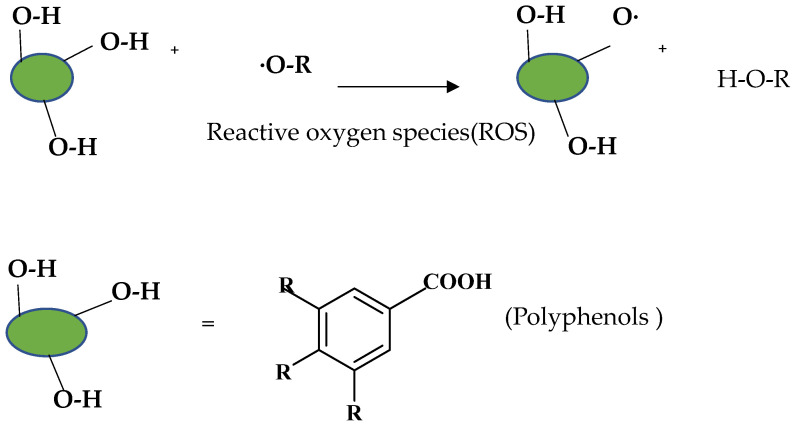
Removal of hydrogen, extinction of ROS, and formation of stabilized polyphenol radicals.

Diet has a great effect on the antioxidant balance of the body. Pathological conditions can occur when the body’s defense mechanisms are destroyed due to nutritional deficiencies [130]. The effects of a diet rich in polyphenolic compounds, which are natural antioxidants, have been examined in various studies, and it has been observed that it reduces oxidative DNA damage [131,132]. As shown in Figure 5, polyphenols react with ROS stoichiometrically to form stabilized radicals [133]. 

Antioxidant activity depends on the existence of hydroxyl groups, which both provide removal of unstable hydrogen and augment the stability of the radical formed.

### 4.1. Phenolic Acids 

Various in vivo and in vitro studies have shown that ferulic acid can reduce cell damage by reducing ROS production and regulating antioxidant enzyme activity, and can also regulate the activation of various cell protective genes against oxidative stress, improve lipid peroxidation and has cardioprotective effects [134]. Aldose reductase (AR) is nicotinamide adenine dinucleotide phosphate (NADPH) as a coenzyme. NADPH is known to play a critical role in the emergence and progression of various oxidative stress-related diseases. Ferulic acid has shown protective effects with its antioxidant and anti-inflammatory properties by acting as an AR inhibitor in the formation of cardiovascular and diabetic complications [68]. The protective effects of gallic acid against oxidative stress appear by regulating lipid peroxidation and NO production and increasing its antioxidant capacity [135]. Gallic acid may be a new generation agent that can be used to combat cellular toxicity caused by arsenic and its metabolite.

### 4.2. Flavonoids

Quercetin is attracting much attention for its potential to prevent cardiovascular, neoplastic and neurodegenerative diseases in which oxidative stress plays a role [136,137,138]. A study investigating the passage of quercetin through the blood–brain barrier (BBB) has shown that oral administration of quercetin to rats exposed to chronic forced swimming alleviates the increased oxidative stress in the brains of the rats through its antioxidant activity [139]. The antioxidative activities of quercetin and catechin were examined in cocoa liquor, and it was stated that quercetin offered higher antioxidant activity than quercetin glycosides [140]. Furthermore, clovamide showed more activity than other components due to the catechin group [140]. The antioxidant properties of coffee have been attributed to both the effect on plasma uric acid and direct absorption of coffee polyphenols [132]. The chlorogenic acid in the composition of coffee showed therapeutic properties by inducing selective anticancer effects in lung cancer cells and leukemia cells at certain concentrations. In contrast, this polyphenolic component has been reported to induce cellular DNA damage at a low millimolar range [141]. 

Delphinidin, an anthocyanidin, has been found to act as a topoisomerase inhibitor in human colon carcinoma cells [142]. In a different study, cyanidin-3-glucoside-rich blackberry extract (at a concentration of 76,000 µg/g) was reported to reduce the growth of HT29 (a human colon cancer cell line widely used in biological and cancer research) cells. In addition, it has been shown that by pre-treatment of HT29 cells with blackberry extract, the complex formation of topoisomerase poisons with DNA is suppressed [143]. Shih et al. (2007) did not detect any toxicity after 24 h of treatment with anthocyanin at 50 µM concentration [144]. Among the anthocyanin compounds they studied, malvidin, cyanidin, kuromanin, delphinidin compounds exhibited higher antioxidant activity. Achieving high expression of phase 2-detoxification enzymes and other antioxidant enzymes was effective in this activity.

### 4.3. Stilbenes

Regarding the cellular defense properties of resveratrol as a direct antioxidant agent, resveratrol has been demonstrated to be a very effective scavenger of a variety of oxidants; the inhibition of ROS/RNS production by intracellular systems exhibited inhibitory effects on the lipid peroxidation and protective effect against protein oxidation intracellular antioxidant defense systems. There are antioxidant defense mechanisms of resveratrol through regulation of signaling pathways. 5′adenosine monophosphate-activated kinase (AMPK) is regulated under metabolic stress and plays an important role in energy homeostasis. Resveratrol increases cell viability against ROS-induced oxidative damage on the myoblast cell line and shows cell protection by enabling AMPK activation [145]. In the case of oxidative stress, resveratrol treatment reduces the protein loss of myoblasts in a dose-dependent manner but cannot prevent the loss completely [146]. In addition, caspase-9 activity, which is a marker of the mitochondrial apoptotic pathway, ceased after 6 h of treatment with resveratrol [146]. Resveratrol exerts a therapeutic effect by activating the protein, an endogenous transcription factor called NRF-2 (the nuclear factor erythroid-derived 2-like), which is associated with antioxidant response [147]. As summarized in Table 3, resveratrol reduces SOD activity and inhibits ROS formation caused by ox-LDL [148].

### 4.4. Lignans 

The effect of flaxseed oil and a flaxseed lignan glucoside derivative in metabolic syndrome disease (characterized by hypertension, increased triglyceride levels, and decreased HDL cholesterol levels) was examined, and it has been determined that oral administration of herbal compounds containing lignans reduces oxidative stress biomarkers such as lipid peroxidation and carbonyl levels [151]. It was stated that the heme oxygenase-1 enzyme, which plays a role in heme catabolism and which is known as a stress-response protein, is induced by the application of lignan-derived compounds, and the lignan derivatives have hepatoprotective and antioxidant properties [152]. Oxidative stress damage in the cerebral cortex recovered in a dose-dependent manner as a result of pretreatment with pinoresinol-4-O-β-D-glucopyranoside, a lignan glycoside has also been reported. It was also noted that this lignan derivative significantly reduced the oxidative stress marker malondialdehyde level and increased catalase activity by about 44% at the specified dose [150,153]. This lignan glycoside is thought to be a potential antioxidant agent in the treatment of hepatic damage, oxidative damage, and diabetes [150]. 

## 5. Polyphenols on DNA and Cancer

In cases where the density of free radicals increases in living cells, structural defects occur in important functional structures such as proteins and nucleic acids. It has been suggested that carcinogenesis, age-related degenerative diseases, and chronic diseases occur when antioxidant defense systems are not sufficiently effective against ROS, which is formed as a result of both biochemical reactions and external factors. In addition, strong findings indicate ROS in both the initiation and the increase in multistage carcinogenesis [154,155].

Oxidative stress causes DNA damage, which triggers multiple cellular responses. Mutations occur by the division of damaged cells that have not been repaired at all or repaired incorrectly. In case of mutations in vital genes such as oncogenes or tumor suppressor genes, cancer initiation and progression processes can be seen [155,156].

The DNA damage response involves the activation of multiple cellular activities that repair DNA lesions and have crucial importance in preventing tumor formation [157]. There is a potential loss of DNA damage repair abilities in the advanced stages of carcinogenesis [155,156]. Cancer cells usually have specific abnormalities in the DNA damage response system; however, chemotherapeutics also have toxicity and persistent side effects on normal cells. With the trial of chemotherapeutics in combination with polyphenols, recent therapeutic strategies such as chemoprevention are gaining importance [158]. 

Polyphenols are among the natural products that are on the agenda as anti-cancer and cancer-curative agents [159]. Dietary polyphenols can influence and modulate many different pathways and processes involved in carcinogenesis. Additionally, they can act as biological response modifiers that support the immune system and protect living cells from damage by free radicals. For example, polyphenolic natural products have anticarcinogenic properties such as modulating cell proliferation, tumor growth, angiogenesis, metastasis, inflammation and apoptosis [16,159]. Polyphenols are reported to protect the body against the effects of reactive oxygen species on DNA integrity but do so reliably only at low concentrations [160].

### 5.1. Phenolic Acids

Ferulic acid may exhibit anticancer properties against tumors that may occur as a result of conditions such as apoptosis mechanisms activated by various endogenous and exogenous pathways [161]. Ferulic acid can prevent oxidative stress from causing permanent damage to any tissue by downregulating related proteins in the mitochondrial apoptosis-promoting protein family [161].

Gallic acid protects against oxidative stress and acute toxicity that may occur in the heart in a study performed on Wistar rats [135]. Thus, it contributes to the protection of kidney and heart health, and it is promised that it can prevent possible cancer formation. Research has shown that gallic acid can inhibit the proliferation of tumor cells in cell cultures by causing apoptosis and/or cell cycle arrest [162]. It has also indicated that the GA induces apoptosis in human lung cancer cells, as well as suppressing DNA damage and provoking DNA repair in prostate cancer cells [163,164].

### 5.2. Flavonoids

In a study involving a large population (over 8000 individuals), high green tea consumption was shown to delay the onset of cancer in patients with premenopausal Stage I and II breast cancer [165]. It has also been found that a lower recurrence rate is observed in breast cancer patients [165]. Ellagitannins, flavonoids, ellagic acid and ellagic acid glycosides in the fruit content show anticancer activity in human colorectal carcinoma cells [166]. 

Epigallocatechin-3-gallate has been shown to provide anticancer activity through many mechanisms such as cell cycle arrest, regulatory role in carcinogenic metabolizing enzymes, inhibition of mitotic signal transduction, antioxidant effect and DNA inhibition. In addition, it regulates the p53 protein, which is one of the transcription factors that regulates the cell cycle [167,168]. 

Naringin’s anti-tumor effects were studied in thyroid cancer as given in Table 4. For 24, 48, or 72 h at 37 °C, Naringin (6, 12, and 25 g/mL) was applied to two cancer cells, TPC-1 and SW1736. In MTT assays, naringin inhibited TPC-1 and SW1736 cell proliferation in a dose- and time-dependent manner [169]. In another study, when Naringin was combined with atorvastatin, it synergistically inhibited prostate cancer cells, PC-3, and LNCaP cells [170].

Quercetin is effective in ovarian cancer by enhancing the antioxidant defense mechanism by suppressing ROS-induced damage [171,172] and in eye cancer by inhibiting the expression of signal proteins produced by hypoxia-induced angiogenesis-stimulating cells [171,172,173]. Furthermore, quercetin shows anticancer properties through different mechanisms in breast, gastric, bone, blood, lung, prostate, brain, skin, colon, thyroid and kidney cancers [174].

In the study of Schantz and coworkers (2010), intracellular ROS level and oxidative DNA damage significantly decreased after 24 h of application of anthocyanin-rich bilberry extract in colon tumor cells [175]. Anthocyanins such as cyanidin and delphinidin in purple tea offer antioxidant, anticancer, and immune system stimulating properties by regulating caspase-3/7 activity and expression levels of certain reductase enzymes [176]. Blueberry anthocyanins and anthocyanin–pyruvic acid adduct inhibited the invasion of matrix-degrading proteinases associated with tumor angiogenesis in breast cancer cell lines [177]. In addition, these two components prevented cell migration by creating a negative chemo-signal and showed an anticarcinogenic effect by preventing metastasis.

Another effective flavonoid, catechin, has anti-cancer properties according to MC38 colon cancer cells [178]. Cancer cell proliferation can be significantly inhibited by catechin concentrations of 250 to 1000 mg/mL, and there is a 50% inhibitory concentration at 250 mg/mL, which is the IC50 value, within an incubation period of 24 h, as given in Table 4. A cell’s death increases levels of triggered cancer cells sharply, and a 72 h incubation time was used with an IC50 value of 142 mg/mL.

### 5.3. Stilbenes

Resveratrol reduces the expression of matrix metalloproteinase, which destroys extracellular matrix components and is responsible for metastasis [179,180,181]. Resveratrol can exhibit both pro-oxidant and antioxidant properties depending on the dose and the cell type it affects [182]. Resveratrol can cause growth arrest and/or cell death in various cancer cells, but its anticancer mechanism of action is not fully known. The anticancer activity of resveratrol has been demonstrated in human colon cancer cells by increasing the level of intracellular ROS and inducing apoptosis through autophagy [183]. Resveratrol presents anticancer properties in lung cancer cells by selectively increasing the expression of a certain group of transmembrane enzymes (NADPH oxidase-5) and thus inducing ROS production [184].

### 5.4. Lignans

The effect of a lignan derivative, schisandrin B, on cardiotoxicity caused by doxorubicin, a cancer chemotherapeutic, was studied, and pretreatment with this compound significantly prevented doxorubicin-induced loss of cardiac function (based on results at 6 and 12 weeks) [185]. Arctigenin induces only inhibition in tumor cells, that is, selectivity between normal and cancerous cells [186]. Another study has concluded that lignan extract induces cytotoxicity, reduces tumor formation in estrogen receptor-positive breast cancer cells, has anti-estrogenic and antioxidant effects, especially at low doses, and may have antitumor components that could prevent chemically induced breast cancer in rats [187]. Some of the research details on the applications of polyphenols on DNA and cancer were presented Table 4.

**Table 4:** Some of the information regarding polyphenols efficacies, working concentration, and target of each substance on DNA and cancer.

Study Design	Outcomes	Ref.
Naringin’s anti-tumor effects on thyroid cancer were studied. For 24, 48, or 72 h at 37 °C, Naringin (6, 12, and 25 g/mL) was applied to two cancer cells, TPC-1 and SW1736.	In MTT assays, naringin inhibited TPC-1 and SW1736 cell proliferation in a dose- and time-dependent manner.	[167]
Naringin was combined with the drug, atorvastatin.	The IC50 of naringin was determined as 196.2 μM in PC-3 and 117.2 μM in LNCaP cells. However, naringin and statin drug atorvastatin synergistically inhibited prostate cancer cells, PC-3, and LNCaP cells.	[168]
Catechin was studied on MC38 colon cancer cells.	The proliferation of cancer cells can be significantly inhibited by catechin concentrations between 250 and 1000 mg/mL, and the IC50 value is 250 mg/mL during a 24 h incubation period. Incubation time was 72 h, and IC50 was 142 mg/mL after triggering cancer cells.	[176]
An anticancer effect of gallic acid on non-small cell lung cancer cells A549.	The viability of A549 cells was determined by MTT assay after treatment with GA (0–52 g/mL) for 24 h. There was a dose-dependent decrease in viability after treatment with GA (0–52 g/mL). When compared with control cells, 12 g/mL GA significantly decreased cell viability.	[188]

## 6. Polyphenols on Aging

ROS plays an important role in the aging process as well as being one of the underlying causes of many chronic diseases [189]. ROS are the normal byproducts of the metabolism of cells responsible for physiological and pathological functions, and considering the dual role of ROS in cells, an antioxidant defense system must be present to neutralize the overproduction of such reactive compounds. The cellular antioxidant defense system consists of endogenous antioxidant enzymes and peptides, as well as polyphenolic compounds, vitamins, and polyunsaturated fatty acids obtained from food sources. The antioxidant defense system gradually deteriorates in time, resulting in oxidative stress and the inability to cope with ROS production, which contributes to the aging process and the onset of age-related diseases. ROS is not only a direct mediator of aging, but also a regulator of the more fundamental aging process [190,191].

Under normal conditions, the skin has highly effective defense mechanisms such as antioxidant enzymes. However, it is known that these mechanisms are insufficient to sustain their effects due to the aging process [192].

In addition to being one of the underlying causes of many chronic diseases, ROS also plays an important role in the aging process [193]. Natural antioxidants are used in pharmaceutical and cosmetic formulations to delay the skin aging process and improve skin aesthetics [194]. 

Due to various physical factors (such as aging, prolonged exposure to UV radiation), a decrease in the number and activities of fibroblast cells is observed in the aging process. Decreased fibroblast activity is accompanied by decreased collagen synthesis. As a result, the activities of collagen proteins to protect the natural structure, moisture and elasticity of the skin are hampered. Wrinkles, roughnesses and hardness appear in the skin, which is seen as “aging” in the physical sense, and natural active ingredients that support fibroblast cell proliferation come to the fore in dermo-cosmetic areas [195,196].

Poplar bud extract containing many polyphenolic compounds such as pinocembrin, galangin, quercetin, kaempferol and apigenin has been studied by creating a special human fibroblast aging model. A two-fold increase in catalase gene expression was found in the group treated with this antioxidant herbal extract [192]. Mohammad et al. (2018) confirmed that the emulsion cream formulation, which also contains bark extract from a natural plant rich in flavonoids, has a skin-rejuvenating effect [197]. This extract also provided increased elasticity and hydration and enriched the skin nutritionally. The fruits of a plant containing polyphenolic components such as chlorogenic acid, catechin quercetin, gallic acid, and cinnamic acid were clinically evaluated, and the tested formulation reduced wrinkles and roughness of the skin by over 10% [198]. Green tea polyphenols were found to greatly support fibroblast cell proliferation and have high moisture retention ability [195]. 

### 6.1. Phenolic Acids

Directly, phenolic acids are antioxidants. Through indirect effects, they stimulate endogenous protective enzymes and signaling pathways. Phenolic acids show antioxidant properties due to the hydroxyl substitution in the aromatic ring and the reactivity of the phenol moiety [24]. In cases where oxidative damage causes tissue degeneration, ferulic acid shows cell proliferation and rapid wound healing properties due to its antioxidant and anti-inflammatory properties [68].

### 6.2. Flavonoids

The antioxidant capacity of anthocyanins varies depending on various modifications such as glycosylation and hydroxylation [199,200]. Among the anthocyanins, it obtained the highest antioxidant activity in the cyanidin-3-glucoside compound, which was even higher than a vitamin E analogue [200]. Auto-fluorescent pigment, an aging marker, was studied to evaluate anti-aging activity [201]. The results showed that plant extract, the main component of which is cyanidin-3-rutinoside, has a strong radical-scavenging effect, reducing the level of endogenous ROS and reducing oxidative stress. Anthocyanins have proven to be effective in the modulation of stress-response genes as well as decreasing ROS accumulation. Anthocyanins have proven to be involved in the modulation of stress-responsive genes, to reduce the accumulation of ROS, to delay aging-related markers, and to show anti-aging activities [201].

Aging is a risk factor for most neurodegenerative diseases that significantly affects quality of life and cognitive levels. The analyses show that anthocyanin might act as an aging suppressor. Anthocyanin was administered to the rats whose aging was accelerated experimentally in order to investigate the effects of anthocyanin on brain aging. When the cognitive and non-cognitive components of behavior were examined after 8 weeks, the use of anthocyanins was shown to reduce DNA damage levels and inflammation accumulation in the brain, prevent age-related cognitive decline and aging, and be a potential approach to maintain thinking and memory. Treatment with 30 mg/kg of anthocyanin improved brain–liver functions in aged model mice with significant deterioration of brain and liver indicators [202]. In addition, atrophy was prevented by preventing changes in brain and liver weight with this treatment. 

To summarize, the anti-aging effects of anthocyanin and its derivatives are: (1) regulation of protein expression associated with DNA damage response; (2) stress-response-related gene expression; (3) stimulating the expression of longevity-related genes [200,201,202]. 

### 6.3. Stilbenes

It has been proven that resveratrol and pterostilbene provide antioxidant and anti-inflammatory effects by activating various signaling pathways and thus have important properties in modulating age-related and chronic diseases [203]. Resveratrol has skin whitening and anti-aging properties. Suppressing or inhibiting the activity of tyrosinase, a limiting enzyme to control melanin production, and ROS scavenging mechanisms play a role in delaying the complications of aging [204]. In a study on flies, the application of resveratrol-rich food alone did not have a lifespan prolonging effect, but it provided improvement in aging-related dysfunctions such as eye degeneration and locomotor disorders [205]. Increased ROS leads to the formation of toxic peroxynitrite and the release of nitric oxide radical, which causes vascular oxidative stress. It was found that increased nitric oxide concentration in cardiomyocytes due to the aging process was significantly reduced even 2 months after resveratrol treatment [206]. Buonocore et al. (2012) applied a nutraceutical 60-day treatment containing phenolic compounds such as resveratrol and procyanidin on 50 healthy middle-aged individuals (who have signs of aging in their skin) [207]. As a result, the antioxidant capacity of the stratum corneum, which is a very important layer of the epidermis, has increased, and skin hardness has been improved [207]. Endothelial dysfunction due to aging is an important risk factor for the development of cardiovascular disease, and alternative treatments are needed. Decreased bioavailability of nitric oxide (NO), vascular damage, autophagy, poor repair processes, and oxidative stress present positive feedback mechanisms. The addition of resveratrol to the treatment in the elderly is reported to be likely to improve vascular functions by causing the regulation of oxidative balance in the endothelium [208].

### 6.4. Lignans

The study with an experimental aging model with rats has concluded that the state of metabolic dysfunction can be regulated with amino acid, lipid and energy metabolism when lignan extract is administered, and that metabolic dysfunction is partially ameliorated by downregulation of some transcription factors. It has been stated that Lignan extract can be used in the prevention and treatment of aging [209].

Superoxide dismutase activity, which is produced as a byproduct of oxygen metabolism and causes cell damage if not regulated, significantly improved after lignan extract treatment. Therefore, it was concluded that lignans can be used clinically as an agent that will exhibit an antiaging function by the regulation of oxygen metabolism and protein expression [210].

Therefore, gel and other kinds of formulations containing the extract of a plant rich in polyphenols can be a promising, anti-aging natural source in the cosmetic field.

## 7. Conclusions and Future Respects 

The prevalence of cardiovascular diseases, oxidative stress-related complications and chronic aging-related ailments is very high and is increasingly common worldwide. These ailments make the lives of patients difficult and in some cases require the use of multiple drugs, and sometimes they cannot be fully treated. It is a fact that when preparing treatment schemes for certain diseases, alternative therapeutic agents with fewer side effects, low toxicity and high patient compliance are needed. By providing appropriate diets, a positive response is obtained against most diseases before the disease is caught or in the early period of the disease. At this point, polyphenolic compounds with antioxidant, anticancer, antidiabetic, anti-inflammatory, and cardiovascular health-protective properties have been the subject of the agenda. Many experimental studies support the view that polyphenols and their sub-group flavonoids slow down the development of atherosclerosis and protect the cardiovascular system from oxidative damage that may result from LDL oxidation. In addition, due to the long-term consumption of foods and beverages with rich polyphenolic content, the formation of cardiovascular diseases is delayed. 

The antioxidant properties of polyphenols are important besides their protective and life-enhancing properties on the heart. There are many studies in the literature that these natural antioxidant compounds reduce the oxidative stress caused by ROS and thereby reduce the risk of cancer formation. In addition, it has been discovered that they can delay the emergence of some diseases by slowing down the aging process that will occur due to many factors such as oxidative stress. Therefore, the importance of consuming foods containing appropriate amounts of polyphenolic compounds should not be underestimated.

In addition to these, the mechanism of action of many polyphenolic bioactive components in the body or which metabolites of these components affect the pathways have not been fully elucidated. Natural antioxidant polyphenols will appear more frequently in the new period experimental studies under the name of nutraceuticals and will take their place in the field as candidates for alternative therapeutic agents.

The polyphenolic contents and amounts for the same compound are different depending on the source. In this review, we examined the effect of certain polyphenols as therapeutic or supportive agents in the treatment of certain diseases. In addition, it was attempted to draw attention to the nutraceutical use of polyphenols by considering their disease-protective effect. However, the positive effects of many polyphenolic structures that have not been researched or entered the literature are waiting to be investigated. One type of feeding should be avoided as much as possible, and diets containing polyphenols should be given more priority. The contents of polyphenolic structures of local plants, which are not well known, should be investigated, and their effects on diseases should be investigated. 

Antioxidant, anti-inflammatory, hypotensive, glucose- and lipid-lowering activities and cardioprotective effects of polyphenols have been demonstrated in many in vivo and in vitro studies. However, some of these observations are also supported by the results of existing clinical trials. One of the reasons for this inconsistency is that polyphenols are extensively metabolized in the human body, affecting their bioavailability. In this regard, it is important to make more modifications and develop new carrier and/or delivery systems to increase the bioavailability of polyphenols. Longer-term clinical studies with rational design and research are needed to examine the therapeutic use of these polyphenols in cardiovascular diseases and to evaluate the safety profile.

## Figures and Tables

**Table 1:** Chemical structure of polyphenols and some examples of subgroups of polyphenols.

Phenolic Acids		Flavonoids	Stilbenes	Lignans
Hydrobenzoic acids 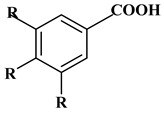	Hydroxycinnamic acids 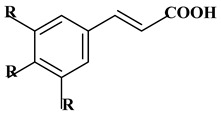	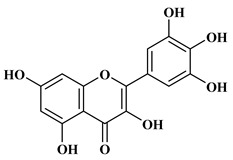	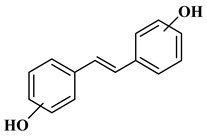	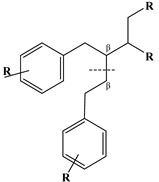
gallic acid 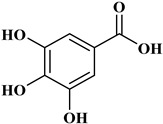	caffeic acid 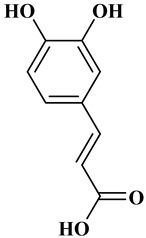	myricetin 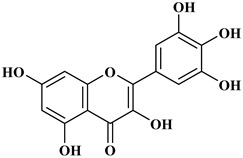	resveratrol 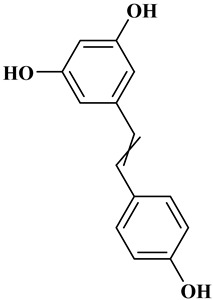	podophyllotoxin 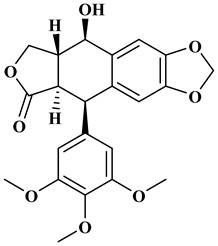
syringic acid 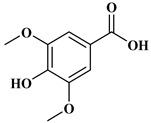	ferulic acid 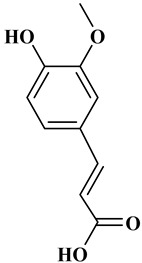	catechin 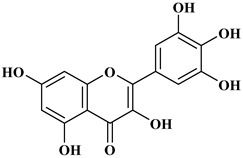	rhapontigenin 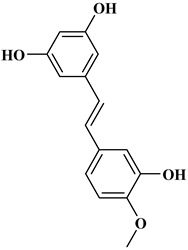	secoisolariciresinol 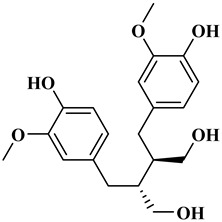
tannic acid 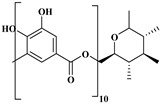	coumaric acid 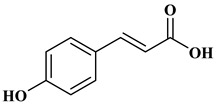	rutin 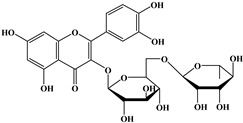	piceatannol 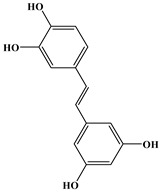	enterodiol 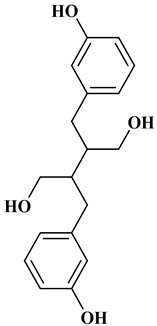
	sinapinic acid 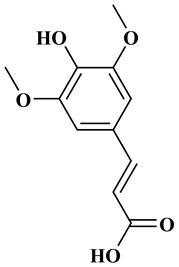	naringin 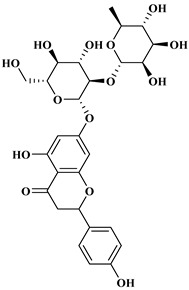	rhaponticin 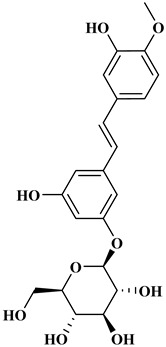	sesamin 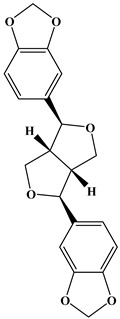
	rosmarinic acid 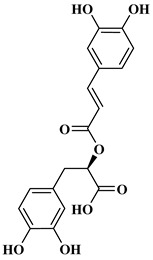	quercetin 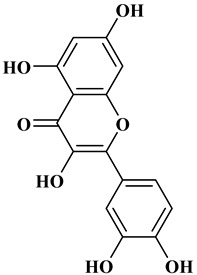	oxyresveratrol 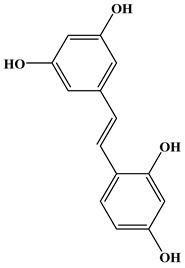	

**Table 3:** Some of the information regarding polyphenols efficacies, working concentration, and target of each substance on ROS-induced oxidative stress.

Study Design	Outcomes	Ref.
A stock solution of resveratrol at a concentration of 200 mg/mL in human umbilical vein endothelial cell (HUVEC); cell culture study	When treated with resveratrol following administration to HUVECs ox LDL, it has been shown that resveratrol inhibits increased ROS formation and lipid peroxidation caused by ox-LDL and reduces SOD activity. Resveratrol effectively inhibited endothelial cell apoptosis through inhibition of mitochondrial pathway and inhibition of oxidative damage. It has been reported that resveratrol may be effective in the treatment of atherosclerosis	[148]
Rosmarinic acid with antioxidant properties with different test methods	RA was determined 67.5 ± 1.68 (μg/mL). Total phenol content in terms of gallic acid equivalency, 1.62 ± 0.41 TEAC value (mM trolox equivalent/g), 806.7 ± 39.5 quercetin eq for total flavonoid test. It was specified 15 μg dry weight RA reduced 366.2 ± 9.9 μmol Fe (III) ions	[32,34]
Administration of resveratrol 50 mg/mL to healthy rats in drinking water for 3 weeks	It shows that resveratrol can improve the capacity of endothelial function and oxidative stress under physiological conditions	[149]
Prunan from prunes alleviates inflammation and oxidative stress during lithium/pilocarpine-induced epileptic seizures	Comparing 50 mg/kg b.w. pinoresinol-4-O-d-glucopyranoside to pilocarpine-treated animals, this compound reduced malondialdehyde levels by 24.2% and increased catalase activity by 44.4%	[150]

## Data Availability

Not applicable.
